# Genomic regulation of invasion by STAT3 in triple negative breast cancer

**DOI:** 10.18632/oncotarget.14153

**Published:** 2016-12-24

**Authors:** Joy M. McDaniel, Katherine E. Varley, Jason Gertz, Daniel S. Savic, Brian S. Roberts, Sarah K. Bailey, Lalita A. Shevde, Ryne C. Ramaker, Brittany N. Lasseigne, Marie K. Kirby, Kimberly M. Newberry, E . Christopher Partridge, Angela L. Jones, Braden Boone, Shawn E. Levy, Patsy G. Oliver, Katherine C. Sexton, William E. Grizzle, Andres Forero, Donald J. Buchsbaum, Sara J. Cooper, Richard M. Myers

**Affiliations:** ^1^ HudsonAlpha Institute for Biotechnology, Huntsville, AL 35806, USA; ^2^ The University of Alabama in Huntsville, Huntsville, AL 35899, USA; ^3^ Department of Oncological Sciences, Huntsman Cancer Institute, University of Utah, Salt Lake City, UT, 84112, USA; ^4^ Department of Pathology, University of Alabama at Birmingham, Birmingham, AL, 35294, USA; ^5^ Department of Radiation Oncology, University of Alabama at Birmingham, Birmingham, AL, 35294, USA; ^6^ University of Alabama at Birmingham Comprehensive Cancer Center, Birmingham, AL 35294, USA; ^7^ Department of Genetics, University of Alabama at Birmingham, Birmingham, AL, 35294, USA

**Keywords:** TNBC, STAT3, ChIP-seq, RNA-seq, invasion

## Abstract

Breast cancer is a heterogeneous disease comprised of four molecular subtypes defined by whether the tumor-originating cells are luminal or basal epithelial cells. Breast cancers arising from the luminal mammary duct often express estrogen receptor (ER), progesterone receptor (PR), and human epidermal growth receptor 2 (HER2). Tumors expressing ER and/or PR are treated with anti-hormonal therapies, while tumors overexpressing HER2 are targeted with monoclonal antibodies. Immunohistochemical detection of ER, PR, and HER2 receptors/proteins is a critical step in breast cancer diagnosis and guided treatment. Breast tumors that do not express these proteins are known as “triple negative breast cancer” (TNBC) and are typically basal-like. TNBCs are the most aggressive subtype, with the highest mortality rates and no targeted therapy, so there is a pressing need to identify important TNBC tumor regulators. The signal transducer and activator of transcription 3 (STAT3) transcription factor has been previously implicated as a constitutively active oncogene in TNBC. However, its direct regulatory gene targets and tumorigenic properties have not been well characterized. By integrating RNA-seq and ChIP-seq data from 2 TNBC tumors and 5 cell lines, we discovered novel gene signatures directly regulated by STAT3 that were enriched for processes involving inflammation, immunity, and invasion in TNBC. Functional analysis revealed that STAT3 has a key role regulating invasion and metastasis, a characteristic often associated with TNBC. Our findings suggest therapies targeting STAT3 may be important for preventing TNBC metastasis.

## INTRODUCTION

Breast cancer is the most prevalent cancer diagnosed in women worldwide, and is the second leading cause of death by cancer in women [1, 2]. In 2015, nearly 231,840 new cases of breast cancer in women were diagnosed in the United States, along with an estimated 40,290 female breast cancer-related deaths [2]. The majority of breast cancers diagnosed are ductal invasive carcinomas. Cancers of this type arise from luminal or basal epithelial cells lining the mammary duct. Ductal-derived breast cancer can be classified into four categories based on microarray gene expression profiling analysis: luminal A, luminal B, human epidermal growth factor receptor 2 (HER2) overexpression, and basal-like (Basal A and Basal B) [3]. Expression of estrogen receptor (ER), progesterone receptor (PR), and HER2 receptor is used to determine the primary breast tumor subtype, prognosis, and targeted therapeutic regimen.

The presence of ER is one of the most important discriminators for diagnosing primary breast tumor subtype [4]. ER-positive tumors are associated with better prognosis because of available targeted hormonal therapy, longer relapse-free survival, and improved overall survival compared to ER-negative tumors [4–6]. While HER2-positive tumors are aggressive, treatment options for these cancers have advanced through the use of monoclonal antibodies to block HER2 activity [7]. Ten to twenty percent of all invasive breast cancers diagnosed are classified as triple negative breast cancer (TNBC), a subtype characterized by a lack of expression of ER or PR, and a lack of HER2 overexpression [8].

TNBCs typically arise from basal cells, and are diagnosed at higher tumor stage and grade, contributing to the aggressive biology of these cancers [8]. TNBC treatment is restricted to the use of cytotoxic chemotherapies because these tumors are non-responsive to anti-hormonal therapeutics [8]. TNBC patients typically receive neoadjuvant chemotherapy, and in comparison to patients with luminal breast cancers, they have a higher initial response to chemotherapy [9, 10]. However, when TNBC and non-TNBC patients are compared longitudinally, TNBC patients have shorter relapse-free survival [9, 10]. TNBC tumors are also more likely to develop resistance to chemotherapies and present with distant recurrence and visceral metastases, all contributing to shorter relapse-free survivals [9–11]. Recurrence typically occurs within 3 years of initial diagnosis [12]. Consequently, in order to advance the development of targeted therapeutics in TNBC, a better understanding of the underlying molecular mechanisms distinguishing TNBC from other breast cancer subtypes is critical.

Genomic studies have reported gene expression signatures characterizing basal TNBC, but lack insight into upstream transcriptional regulators [3, 13, 14]. The high success rate of targeting the transcription factor ER in luminal breast cancers supports the notion that identifying the transcriptional regulator(s) of basal TNBC will be beneficial in the development of therapies for this aggressive breast cancer. Key transcription factors as potential therapeutic targets in basal TNBC are likely to be overexpressed in TNBCs compared to ER+ tumors, and possess known oncogenic mechanisms in other solid tumors. One transcription factor that meets these criteria as a potential therapeutic target in TNBC is signal transducer and activator of transcription 3 (STAT3).

STAT3 has been widely recognized as an oncogene in various cancers and has been confirmed to be constitutively active in TNBC [15, 16]. There is no difference in the gene expression levels of STAT3 in ER+, HER2, and TNBC breast cancer subtypes; however, active phosphorylated STAT3 is restricted to basal TNBCs [17, 18]. Furthermore, it has been shown that STAT3 signaling is critical for cell survival in basal TNBC breast tumors [18]. As an oncogene, in other cancer types, STAT3 has been shown to regulate various aspects of cancer onset and progression including transformation, proliferation, invasion, and metastasis [16, 19, 20]. Treatment of TNBC cell lines with pharmacological agents targeting STAT3, revealed this factor to be required for cancer stem cell maintenance and cell survival in TNBC [15, 17, 18, 21]. These studies implicate a role for STAT3 in the aggressive biology of TNBC; however the transcriptional program associated with STAT3 and its molecular mechanisms are unknown. Here we describe our efforts to characterize the direct regulatory gene targets of STAT3 and its functional role in the progression of basal TNBC.

## RESULTS

### Genome-wide binding patterns of STAT3

**Figure 1:**
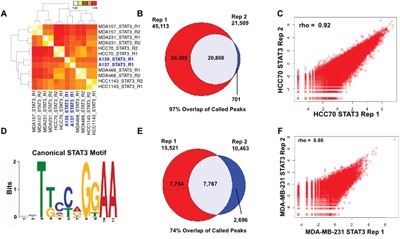
STAT3 binds in a subtype-specific manner across basal TNBC breast cancer **A.** Heatmap of Spearman rank correlations between all pairwise comparisons of STAT3 binding sites across basal TNBC cell lines and TNBC tumors (blue). **B.** Venn diagram of replicate STAT3 binding sites in basal breast cancer cell line HCC70. 20,808 high-confidence binding sites were called in both replicates. **C.** Correlation plot of STAT3 binding sites in HCC70 replicates (Spearman rho=0.92) **D.** Canonical STAT3 motif enriched in binding sites in HCC70 and MDA-MB-231. **E.** Venn diagram of replicate STAT3 binding sites in basal breast cancer cell line MDA-MB-231. 7,767 high-confidence binding sites were called in both replicates. **F.** Correlation plot of STAT3 binding sites in MDA-MB-231 replicates (Spearman, rho=0.80).

To characterize STAT3 binding patterns associated with TNBC, we performed, in replicate, Chromatin Immunoprecipitation followed by massively parallel sequencing (ChIP-seq) in five basal breast cancer cell lines with a STAT3-specific antibody [22]. We identified genomic regions commonly as well as uniquely bound by STAT3 across the breast cancer cell lines. Hierarchical clustering of STAT3 binding sites demonstrated that STAT3 binding is heterogeneous, possibly reflecting the heterogeneity of TNBC (Figure 1). We next evaluated gene enrichment at TNBC-specific STAT3 binding sites using the Genomic Regions Enrichment of Annotations Tool (GREAT) [23], and found these sites were near genes down-regulated in luminal breast cancer and up-regulated in basal breast cancer cell lines (Supplementary Table S1). Notably, these TNBC-bound STAT3 sites were further enriched near genes in pathways involved in cell migration and invasion, including extracellular matrix organization, extracellular structure organization, collagen metabolic process, anchoring junctions, adherens junctions and regulation of locomotion [23] (Supplementary Tables S2-S3). These enriched pathways are related to invasion, and these findings point to a role for STAT3 in this key tumorigenic process in TNBCs.

### Comparison of STAT3 binding in cell lines with patient-derived tumor samples

Because human breast cancer cell line models were used to characterize the genome-wide binding patterns of STAT3 in TNBC, we next determined if these genome-wide binding patterns are also seen in primary tissue from TNBC tumors. We performed ChIP-seq on STAT3 in two frozen TNBC patient tumors [24], and identified 9,074 and 12,780 STAT3 binding events. We observed that STAT3 binding in TNBC tumor tissues is highly correlated with binding sites identified in basal TNBC cell lines (Figure 1). Similarly, STAT3 binding sites in tumors and cell lines are enriched for biological processes involving invasion mechanisms (Supplementary Figure S1). Importantly, these data indicate STAT3 cell line binding sites are also seen *in vivo* in patient tumors.

The high similarity of STAT3 binding patterns in TNBC tumors and cell lines led us to further analyze the binding patterns of STAT3 in two TNBC cell lines. Due to the heterogeneity of TNBC breast cancers, we selected two cell lines harboring broad molecular subtypes of TNBC-basal A (HCC70) and basal B (MDA-MB-231) tumors. ChIP-seq binding sites for each cell line was integrated with RNA-seq data to determine STAT3 driven gene regulation in TNBC. In HCC70, 20,808 high-confidence STAT3 binding sites were identified (Figure 1). Pairwise analysis of each ChIP-seq replicate revealed a significant correlation of binding sites across replicates (Spearman, rho=0.92) (Figure 1). These reproducible binding sites were significantly enriched for the canonical STAT3 motif (Figure 1D. Similarly, in the basal B cell line (MDA-MB-231), there were 7,767 high-confidence binding sites in both replicates (Figure 1). Again, these binding sites had both a high correlation coefficient (Spearman, rho=0.80), and a significant enrichment of the canonical STAT3 motif (Figure 1). The high-confidence binding sites for both cells lines were used for integration with RNA-seq data to characterize the effects of STAT3 binding on gene expression.

### Effects of STAT3 on gene expression in TNBC cell lines

**Figure 2:**
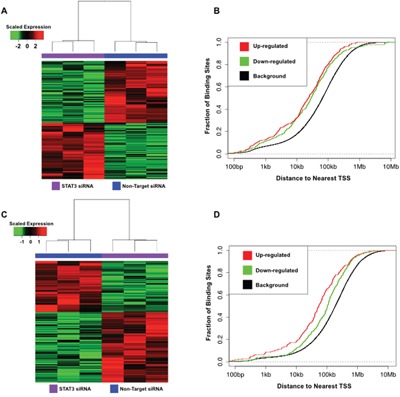
STAT3 binds and regulates genes in basal TNBC cell lines **A.** Heatmap of 737 differentially expressed gene transcripts in HCC70 in response to STAT3 knockdown (> 2.0-fold differences between siRNA treated cells and non-targeting vehicle controls) **B.** Cumulative distribution function plot of STAT3 binding sites near differentially expressed transcripts in HCC70. **C.** Heatmap of 548 differentially expressed gene transcripts in MDA-MB-231 in response to STAT3 knockdown (> 2.0-fold differences between siRNA treated cells and non-targeting vehicle controls) **D.** Cumulative distribution function plot of STAT3 binding sites near differentially expressed transcripts in MDA-MB-231.

To ascertain which genes are regulated by STAT3 in these basal TNBC cell lines, we performed RNA-seq under control culture conditions and after 96 hour siRNA-mediated knockdown of STAT3 (Supplementary Figure S2) [25]. Analysis of the RNA-seq data revealed 737 differentially expressed transcripts in HCC70 (Figure 2) and 548 differentially expressed transcripts in MDA-MB-231 (Figure 2) (> 2.0-fold differences between siRNA treated cells and non-targeting vehicle controls).

Integration of the RNA-seq and STAT3 ChIP-seq datasets in HCC70 cells showed that 50% of the differentially regulated transcripts (245 transcripts down-regulated and 231 transcripts up-regulated; p-value < 2.22 × 10^-16^ and p-value < 5.64 × 10^-14^, respectively, k-s test) have a STAT3 binding site within 50 kb of their transcriptional start site (TSS) (Figure 2). These differentially expressed transcripts within 50 kb of a TSS are likely regulated by STAT3 in HCC70. Similarly in the MDA-MB-231 cell line, 216 genes (104 transcripts down-regulated and 112 transcripts up-regulated; p-value < 8.07 × 10^-09^ and p-value < 1 × 10^-16^, respectively, k-s test) were identified to have a STAT3 binding site within 50 kb of the gene's TSS (Figure 2). In each condition, STAT3 binding sites were significantly closer to differentially expressed gene TSSs than random transcripts, which is consistent with regulation of gene expression. These findings suggest STAT3 acts more as a distal regulator in the context of TNBC, supporting previous findings of STAT3 distal gene regulation during normal development and in disease states [26].

### STAT3 regulates genes involved in cellular invasion and migration processes

Discovery of STAT3 transcriptional activity in TNBC led us to assess the transcripts commonly regulated by this factor in both TNBC cell lines. We identified 22 common transcripts likely regulated by STAT3 (Supplementary Table S4). This small signature was not enriched for any biological processes; however, it provides insight to the potential regulatory mechanisms of STAT3 in TNBC. Interestingly, these findings indicate that STAT3 transcriptional activation in basal TNBC is heterogeneous, consistent with the heterogeneity of the phenotypes of TNBC in different patients. Therefore, we analyzed the direct target signatures unique to each cell line to discover pathways likely regulated by STAT3 in TNBC subtypes.

In the previously described HCC70 transcript signature, we discovered pathway enrichments activated by STAT3 (down-regulated after STAT3 knockdown) to include matrisome (FDR=7.85×10^-12^), inflammatory response (FDR=2.51×10^-04^), and immunity (FDR=1.26×10^-03^) (Table 1). By contrast, pathway enrichment for transcripts likely repressed by STAT3 (up-regulated upon STAT3 knockdown) include matrisome (FDR=2.96×10^-04^), secreted factors (FDR=1.10×10^-07^), and epithelial to mesenchymal transition (EMT) (FDR=1.17×10^-05^) (Table 1). In MDA-MB-231, pathway enrichments for transcripts likely activated by STAT3 included G-protein coupled receptor (GPCR) ligand binding (FDR=2.59×10^-04^), matrisome (FDR=8.54×10^-04^), and complement and coagulation cascades (FDR=8.59×10^-04^) (Table 2). Similar to HCC70, STAT3 in MDA-MB-231 was found likely to repress pathways enriched for transcripts involving secreted factors (FDR=2.03×10^-06^), matrisome (FDR=9.18×10^-06^), and matrisome associated (FDR=2.64×10^-04^) (Table 2). We also performed STAT3 ChIP-seq and RNA-seq (following STAT3 knockdown) in three additional cell lines (Supplementary Figures S2-S4). Integration of these datasets reveals significant enrichments for invasion processes (Supplementary Tables S5-S7). These results further confirm a key role for STAT3 as a regulator of invasion in TNBC. The enrichments are relevant because TNBC subtypes are likely to be invasive and metastatic, contributing to the aggressive nature of these cancers. STAT3 may play an important role in remodeling the extracellular matrix, exploiting inflammatory mechanisms, and evading immune surveillance to create a metastatic niche to facilitate spread of the cancer.

**Table 1:** Top 10 GSEA enrichments of differentially expressed genes in response to 96 hour STAT3 knockdown in HCC70

Signatures Directly Activated by STAT3 in HCC70 (Down-regulated Upon STAT3 Knockdown)	FDR q-value
NABA_MATRISOME	7.85E-12
NABA_MATRISOME_ASSOCIATED	1.46E-06
HALLMARK_INFLAMMATORY_RESPONSE	2.51E-04
NABA_CORE_MATRISOME	2.51E-04
NABA_ECM_REGULATORS	6.92E-04
KEGG_COMPLEMENT_AND_COAGULATION_CASCADES	1.17E-03
REACTOME_IMMUNE_SYSTEM	1.26E-03
NABA_ECM_GLYCOPROTEINS	1.26E-03
REACTOME_REGULATION_OF_COMPLEMENT_CASCADE	2.42E-03
BIOCARTA_IL22BP_PATHWAY	3.33E-03
**Signatures Directly Repressed by STAT3 in HCC70 (Up-regulated Upon STAT3 Knockdown)**	**FDR q-value**
NABA_MATRISOME	2.96E-14
NABA_MATRISOME_ASSOCIATED	1.30E-10
NABA_SECRETED_FACTORS	1.10E-07
HALLMARK_EPITHELIAL_MESENCHYMAL_TRANSITION	1.17E-05
REACTOME_CLASS_A1_RHODOPSIN_LIKE_RECEPTORS	3.13E-05
REACTOME_GPCR_LIGAND_BINDING	4.27E-05
REACTOME_PEPTIDE_LIGAND_BINDING_RECEPTORS	6.05E-05
HALLMARK_COAGULATION	9.12E-05
HALLMARK_INFLAMMATORY_RESPONSE	8.62E-04
HALLMARK_TNFA_SIGNALING_VIA_NFKB	8.62E-04

Enrichments represent hallmark and canonical pathways in GSEA database. STAT3 responsive genes are enriched for processes involving invasion

**Table 2:** Top 10 GSEA enrichments of differentially expressed genes in response to 96 hour STAT3 knockdown in MDA-MB-231

Signatures Directly Activated by STAT3 in MDA-MB-231 (Down-regulated Upon STAT3 Knockdown)	FDR q-value
REACTOME_GPCR_LIGAND_BINDING	2.59E-04
REACTOME_CLASS_A1_RHODOPSIN_LIKE_RECEPTORS	2.77E-04
BIOCARTA_CLASSIC_PATHWAY	5.36E-04
BIOCARTA_COMP_PATHWAY	8.54E-04
NABA_MATRISOME	8.54E-04
KEGG_COMPLEMENT_AND_COAGULATION_CASCADES	8.59E-04
KEGG_HEMATOPOIETIC_CELL_LINEAGE	1.94E-03
REACTOME_G_ALPHA_WE_SIGNALLING_EVENTS	2.03E-03
REACTOME_GPCR_DOWNSTREAM_SIGNALING	4.23E-03
NABA_ECM_REGULATORS	4.23E-03
**Signatures Directly Repressed by STAT3 in MDA-MB-231 (Up-regulated Upon STAT3 Knockdown)**	**FDR q-value**
NABA_SECRETED_FACTORS	2.03E-06
HALLMARK_HYPOXIA	9.18E-06
NABA_MATRISOME	9.18E-06
KEGG_CYTOKINE_CYTOKINE_RECEPTOR_INTERACTION	3.72E-05
HALLMARK_TNFA_SIGNALING_VIA_NFKB	1.16E-04
NABA_MATRISOME_ASSOCIATED	2.63E-04
REACTOME_ACTIVATION_OF_GENES_BY_ATF4	1.35E-03
REACTOME_PERK_REGULATED_GENE_EXPRESSION	1.52E-03
HALLMARK_INFLAMMATORY_RESPONSE	1.52E-03
HALLMARK_UNFOLDED_PROTEIN_RESPONSE	2.88E-03

Enrichments represent hallmark and canonical pathways in GSEA database. STAT3 responsive genes are enriched for processes involving invasion

### Cellular invasion assays support a role for STAT3 in regulating invasion in TNBC

**Figure 3:**
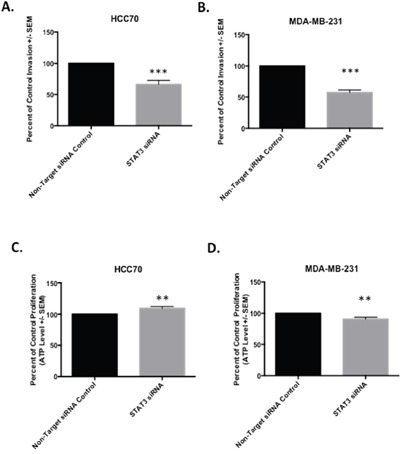
STAT3 regulates a gene signature associated with an invasion phenotype in basal TNBC (Data represents mean +/− SEM) **A.** Transwell invasion after STAT3 knockdown in HCC70 resulted in 1.6-fold reduced invasion (t-test, p-value<0.0001). **B.** Transwell invasion after STAT3 knockdown in MDA-MB-231 resulted in 1.9-fold reduced invasion (t-test, p-value < 0.0001). **C.** HCC70 proliferation measure after siRNA mediated STAT3 knockdown for 96 hrs. Knockdown of STAT3 did not result in reduced proliferation, but increased proliferation by 10% (t-test, p-value<0.0037). **D.** MDA-MB-231 proliferation measure after STAT3 knockdown for 96hrs. Knockdown of STAT3 resulted in 10% decrease in proliferation (t-test, p-value < 0.0040).

Our results suggest a potential role for STAT3 in cellular invasion and migration in TNBC. We further investigated its role in invasion in HCC70 and MDA-MB-231 cells by siRNA-mediated knockdown of STAT3 followed by matrigel transwell invasion assays. We observed a significant 44%(t-test, p-value<0.0001) and 42% decrease (t-test, p-value<0.0001) in invasion of HCC70 and MDA-MB-231 cells, respectively transfected with the STAT3 siRNA pool (Figure 3). The significant reduction of invasive potential in both cell lines reveals a functional regulatory role for STAT3 in the invasive properties characteristic of TNBCs. To ensure that STAT3 knockdown did not simply result in reduced overall viability of the cells, we also performed cellular proliferation assays, but did not observe significant differences in the viability of HCC70 or MDA-MB-231 cells, even after 96 hours of STAT3 knockdown treatment (Figure 3). Collectively, these data support a unique role for STAT3 in cellular invasion and migration.

## DISCUSSION

This work reveals the transcriptional program regulated by STAT3 in TNBC and its enrichment for invasion related processes. Invasion involves the intravasation of cancer cells from the primary tumor location into the vasculature; this is followed by cell metastasis to the distal tumor site [27–29]. It is well established that TNBC tumors are metastatic and chemoresistant in nature, which are contributing factors to the lack of targeted therapy for this breast cancer subtype. Various gene expression studies have characterized the genes expressed in basal TNBC, distinguishing this cancer from other breast cancer subtypes. These TNBC specific genes contribute to the aggressive biology of this cancer; however, the upstream transcriptional regulators remain elusive. To facilitate targeted therapy, the upstream transcriptional regulators of TNBC gene signatures must be identified.

Our studies revealed STAT3 as a key regulator of basal TNBC. STAT3 is constitutively activated in basal TNBCs; however, full characterization of its genomic regulatory mechanisms is undetermined. We conducted ChIP-seq to characterize the genome-wide binding patterns of STAT3 in TNBC tumors and cell lines. STAT3 binding in cell lines and tumors was enriched near pathways involving invasion, such as extracellular matrix remodeling and cell migration. From these results, we inferred that STAT3 appears to be involved in the regulation of invasion mechanisms in TNBC.

Integration of RNA-seq data from TNBC cell lines, after knockdown of STAT3, allowed for the prediction of genes likely regulated by STAT3. We found that genes potentially regulated by STAT3 were mostly cell line specific, further corroborating the known heterogeneity of basal TNBC. Despite little overlap of genes likely regulated by STAT3 in the cell lines, the most significantly enriched process in both cell lines was for matrisome (the ensemble of extracellular matrix (ECM) proteins and related ECM proteins) formation [30, 31]. Matrisome processes were activated and repressed by STAT3, and it is likely that STAT3 promotes the degradation of the ECM and inhibits its maintenance to promote invasion. The TNBC matrisome was indeed impacted by STAT3 perturbation, as the transwell assays allowed us to functionally test regulation of the matrisome by STAT3. Knockdown of STAT3 resulted in decreased invasive potential of the basal TNBC cell lines across matrigel-coated wells. Taken together, STAT3 regulates pro-invasion and pro-metastatic gene signatures in a TNBC subtype specific manner.

STAT3 has been implicated in various cancers, including breast, ovarian, prostate, and lung cancers, as having a regulatory role in cancer development, including transformation, proliferation, EMT, invasion, and metastasis [16]. However, until now, the role of STAT3 in the context of basal TNBC was unknown. Recent studies revealed that phosphorylated STAT3 is associated with genes involved in immunity, inflammation, and invasion in TNBC transcriptomic data from TCGA [32]. These findings further supported a key regulatory role for STAT3 in TNBC. However, it did not describe direct gene signatures regulated by STAT3, nor functional characterization of the role of STAT3 in invasion in TNBC. Our work builds on this study as we further uncovered gene signatures likely regulated by STAT3 that corroborates the functional enrichments described by independent studies [32]. Moreover, we provide functional data to support a role for STAT3 in the regulation of cellular invasion processes in TNBC. While the direct gene targets of STAT3 are subtype specific across TNBC, indicative of the heterogeneity of this disease, our findings suggest this factor plays a universal role of regulating invasion in this cancer.

Further investigation must be conducted to unravel the role of STAT3 in actual metastasis of TNBC tumor cells to a secondary site. Development of TNBC xenograft mouse models followed by STAT3 perturbation will assist in discovering if STAT3 is indeed a therapeutic target for inhibiting metastasis of TNBC tumors. Direct inhibition of STAT3 may potentially improve chemotherapy response and decrease metastasis in TNBC, ultimately improving overall survival of TNBC patients. STAT3 subtype specific gene signatures could serve as potential biomarkers for determining the specific subtype of TNBC tumor and appropriate choice of STAT3 inhibition and chemotherapy for personalized treatment. Additional studies aimed at understanding the pharmacological response of the TNBC subtype specific STAT3 targets are needed to develop personalized therapies for inhibiting this factor.

## MATERIALS AND METHODS

### Cell culture and harvest

The following cell lines: MDA-MB-231, MDA-MB-157, MDA-MB-468 were cultured in DMEM media (Gibco, ThermoFisher Scientific, Waltham, MA) supplemented with 10% fetal bovine serum (Hyclone, GE Healthcare, Logan, UT), 1 mM sodium pyruvate, and non-essential amino acids (Gibco). HCC70 and HCC1143 were cultured in RPMI media (Gibco), supplemented with 10% fetal bovine serum (Hyclone), 10 mM HEPES (Sigma-Aldrich, St. Louis, MO), 1 mM sodium pyruvate (Gibco), and 4.5 g/l glucose (Sigma-Aldrich). Cells were passaged and harvested at 80% confluence. Prior to harvest, cells were treated for 72hr or 96hr with either 10 μM STAT3 siRNA or an equal concentration of control non-targeting siRNA.

For ChIP-seq experiments, protein-DNA complexes were covalently cross-linked by incubating cells in 1% formaldehyde for 10 minutes at room temperature. Cells were incubated with 0.125 M glycine for 5 minutes, to quench cross-linking reaction. Cells were washed and scraped with PBS (pH 7.4) (Lonza, Walkersville, MD). Cells were lysed with Farnham Lysis Buffer (5mM PIPES at pH 8.0, 85 mM KCl, 0.5% NP-40) containing protease inhibitor (Roche Diagnostics, Indianapolis, IN). Cell lysate was centrifuged at 2000 rpm for 5 minutes at 4°C. The crude nuclear extract contained in the supernatant was stored at -80°C.

For RNA-seq, cells were treated with Buffer RL (Norgen Biotek Corporation, Thorold, ON, CAN) and stored at -80°C. Cell lysate was collected after addition of 100% molecular biology grade ethanol. Total RNA was extracted using the Norgen Animal Tissue RNA Purification Kit.

### RNAi knockdown of STAT3

We used an ON-TARGETplus Human STAT3 siRNA kit from GEHealthcare (L-003544-00-0005, GE Healthcare, Logan, UT). This SMARTpool siRNA contains four pooled siRNAs, each targeting a separate region of the STAT3 RNA sequence. We also used ON-TARGETplus Non-targeting siRNA #1 (D-001810-01-05) as a non-targeting control. The siRNA SMARTpool and Non-targeting siRNA target sequences are below:

ON-TARGETplus SMARTpool siRNA J-003544-07, STAT3

Target Sequence: GAGAUUGACCAGCAGUAUA

ON-TARGETplus SMARTpool siRNA J-003544-08, STAT3

Target Sequence: CAACAUGUCAUUUGCUGAA

ON-TARGETplus SMARTpool siRNA J-003544-09, STAT3

Target Sequence: CCAACAAUCCCAAGAAUGU

ON-TARGETplus SMARTpool siRNA J-003544-10, STAT3

Target Sequence: CAACAGAUUGCCUGCAUUG

ON-TARGETplus Non-targeting siRNA #1

Target Sequence: UGGUUUACAUGUCGACUAA

The siRNA transfection experiments were performed in 6-well and 96-well cell culture plates in triplicate and quintuplicate, respectively, and included the non-targeting control. For transfection, the Lipofectamine RNAiMAX Transfection Reagent (ThermoFisher Scientific) was used. Lipofectamine and siRNAs were prepared per manufacturer's instructions (ThermoFisher Scientific). To each well containing cells, in the 96-well plate experiments (viability), we added 10 ul of the siRNA-transfection reagent mix diluted in Opti-MEM I Reduced Serum Medium (ThermoFisher Scientific); this resulted in 1 pmol siRNA in 0.3 μl Lipofectamine RNAiMAX reagent per well. For 6-well cell culture plate experiments, 250 μl siRNA-transfection reagent mix was added to each well, for a final concentration of 25 pmol siRNA in 7.5 μl Lipofectamine RNAiMAX reagent per well.

### SDS-PAGE and western blotting

To confirm knockdown of STAT3 protein levels, SDS-PAGE followed by western blotting was performed. HCC70 and MDA-MB-231 cells were seeded in six-well plates, at a density of 300,000 (MDA-MB-231) or 600,000 (HCC70) cells per well. After 96 hours, cells were harvested via trypsinization followed by centrifugation at 2,000 rpm for 5 minutes. Cell pellets were washed in 1 mL PBS, centrifuged a second time, and stored at -80°C until immunoprecipitation step. Cell pellets were resuspended in 100 μl RIPA buffer containing protease inhibitors, and incubated, on ice, for 30 minutes. Cells were dounced with a syringe to further lyse cells, followed by centrifugation at 14,000 rpm for 10 minutes. The supernatant was collected and protein concentrations were measured using the Qubit Protein Assay kit and Qubit fluorometer (ThermoFisher Scientific). 40 μg protein for each cell line and condition were mixed with an equal volume of Laemelli Buffer (Bio-Rad, Hercules, CA) supplemented with 10% Beta-mercaptoethanol (Sigma). Protein samples were heated in a 95-100°C water bath for ten minutes. Protein lysate samples and SeeBlue Plus2 (ThermoFisher Scientific) and Magic Mark XP (ThermoFisher Scientific) protein standards were loaded onto a pre-cast SDS-polyacrylamide gel (Bio-Rad) assembled in a reservoir containing 1X Tris/Glycine buffer (BioRad).

The SDS-PAGE gel was run for 1.5 hours at 150V. Proteins were transferred to a nitrocellulose membrane using the iBlot gel transfer device, per the manufacturer's instructions, using program 3 for seven minutes (ThermoFisher Scientific). Following transfer, the protein-containing membrane was placed in blocking reagent (PBS + NF dairy milk + 10% Tween), followed by shaking for 1 hour at room temperature. Blocking reagent was discarded and wash buffer containing antibodies targeting STAT3 (Santa-Cruz) phosphorylated STAT3 (Ser727) (Cell Signaling Technology), β-actin (loading control) (Cell Signaling Technology) were added to the membrane and incubated overnight at 4°C, with rotation. The membrane was washed three times, for ten minutes each, with western wash buffer (1X PBS with 0.05% Tween 20). Wash buffer containing anti-rabbit or anti-mouse secondary antibody was added to the membrane and incubated for 1 hour at room temperature. The membrane was again, washed three times for ten minutes. To visualize blotted proteins, SuperSignal West Femto working solution was prepared by mixing equal parts of stable peroxide and luminol/enhancer, per manufacturer's instructions (ThermoFisher Scientific) and incubated with the membrane for 5 minutes. Imaging was conducted using the UVP imaging system.

### Matrigel invasion assays

To determine if STAT3 regulates invasion, 300,000 (HCC70) or 75,000 (MDA-MB-231) cells were seeded into six-well plates and transfected with STAT3 siRNAs and non-targeting siRNAs as described above. Cells were incubated for 72 hours (HCC70) or 96 hours (MDA-MB-231) at 37°C/5%CO_2_. Cells were harvested via trypsinization followed by centrifugation at 2,000 rpm for five minutes. Cell pellets were resuspended in 10 ml media and quantified using a hemocytometer. Cells were diluted to a concentration of 40,000 cells/ml in serum free media and seeded onto Corning Biocoat matrigel invasion chambers (Corning, Bedford, MA) as previously described [33]. Student's t-test was applied to calculate significance using GraphPad Prism (GraphPad Software, La Jolla, CA).

### Cell viability assays

Cell viability assays were conducted in 96-well cell culture plates. HCC70 and MDA-MB-231 cells were transfected with a siRNA targeting STAT3 or a non-targeting siRNA vehicle control for 96 hours. Cell proliferation was measured using the ATPLite Assay (Perkin Elmer, Waltham, MA) for cell metabolism.

### ChIP-seq

To characterize genome-wide binding patterns of STAT3 in TNBC cell lines, ChIP-seq was performed as previously described [34]. Antibodies for STAT3 (Santa Cruz, sc-482, Santa Cruz Biotechnology, Santa Cruz, CA) were used. Replicate experiments with high similarity (> 80% concordance across called peaks), were combined into a single data set of high confidence binding sites.

Measurement of STAT3 binding in TNBC tumor tissues was performed as previously described [24].

### RNA-seq

Cells were lysed with Buffer RL (Norgen) containing 10% beta-mercaptoethanol. Cell lysate was collected after addition of 300 μl 100% molecular biology grade ethanol. Total RNA was extracted using the Animal Tissue RNA Purification Kit (Norgen). RNA-seq libraries were prepared from 250 ng total RNA via polyA-selection (Dynabead mRNA Purification Kit, ThermoFisher Scientific) followed by transposase-mediated non-stranded library construction [25]. Each experimental treatment was performed in triplicate. Libraries were pooled and sequenced on an Illumina HiSeq 2000 sequencer using paired-end 50 bp reads with a 6 bp index read (Illumina, Inc., San Diego, CA). Pooled sequencing resulted in 26.5 million reads per library. Differential expression was measured using the DESeq2 program [35]. TopHat (version 1.4.1) was used to align RNA-seq paired reads to GENCODE (version 9.0) [36, 37]. Cufflinks (version 1.3.0) and BEDTools [38, 39] were used to calculate raw counts for each GENCODE transcript. For this study X and Y chromosome transcripts were omitted.

### Integrated genomic analysis

For each ChIP-seq replicate, peaks were identified and reported using the MACS peak caller program [40]. Motif analysis for binding sites identified in individual replicates was performed using MEME [41]. After identification of STAT3 binding sites, the normalized read depth for each experiment was calculated by tabulating the number of reads across binding sites and normalizing the reads to the total number of aligned reads generated for each experiment. The normalized reads were mapped by centering each ChIP-seq peak summit around 100bp sequences. Spearman correlation coefficients were calculated across normalized STAT3 binding sites and clustered to identify differential STAT3 binding patterns across basal tumors and cell lines. STAT3 peaks common in TNBC cell lines and tumors were analyzed for gene ontology enrichment using the GREAT program [23]. In HCC70 and MDA-MB-231 ChIP-seq studies, only reproducible binding sites identified in replicates, were called as high-confidence bindings sites and used for integrated analysis with RNA-seq data sets.

Differential gene expression analysis was performed in the statistical software, R (version 3.2.1) [42], using DESeq2 (version 1.8.1), using the default settings [35]. Differentially expressed transcripts were filtered for only those transcripts with > 2.0 fold differences between STAT3 siRNA treated cells and non-targeting siRNA vehicle controls. Differentially expressed transcripts were normalized, followed by hierarchical clustering using the hclust command in R (method=”ward”, distance=”euclidean”). Differential transcripts were integrated with high confidence ChIP-seq binding sites and the central distribution function was calculated to predict what fraction of significant transcripts have a STAT3 binding site within 50 kb of the TSS. 50kb was chosen as a cutoff based on data suggesting that the correlation between TF binding and gene expression tails off beyond about 100kb [43]. The Komolgorov-Smirov test (KS-test) was implemented to determine the significant enrichment of STAT3 binding near differentially expressed transcripts compared to the background genome using the ks.test command in R.

Differentially expressed genes with a nearby binding site were considered to be direct targets and were used for Hallmark and Canonical Pathway enrichments using Gene Set Enrichment Analysis [44, 45]. Student's t-tests were used to calculate significance of cell viability and invasion assays.

All ChIP-seq and RNA-seq datasets have been deposited in GEO (accession number: GSE85579).

## SUPPLEMENTARY MATERIALS FIGURES AND TABLES



## Abbreviations

ChIP-seq: Chromatin Immunoprecipitation followed by massively parallel sequencing
ECM: Extracellular Matrix
ER: Estrogen Receptor
HER2: Human Epidermal Growth Factor Receptor 2
PR: Progesterone Receptor
STAT3: Signal Transducer and Activator of Transcription 3
TF(s): Transcription Factor(s)
TNBC: Triple Negative Breast Cancer
Acquisition of data: JMM, KEV, JG, BSR, DSS, KMN, SKB, LAS, ECP, ALJ, BB, SEL and RMM
Analysis and interpretation of data: JMM, DSS, BSR, RCR, SKB, LAS, SJC and RMM
Writing, review and/or revision of manuscript: JMM, DSS, BNL, MKK, RCR, LAS, DJB and RMM
Administrative, technical or material support: PGO, KCS, AF, and WEG
