# Supplementary material for: Genomic regulation of invasion by STAT3 in triple negative breast cancer
Source: Oncotarget. 2016 Dec 24;8(5):8226–38. doi: 10.18632/oncotarget.14153 (PMC5352396; doi:10.18632/oncotarget.14153)
Supplement: Supplementary file 1 [file oncotarget-08-8226-s001.pdf]

Genomic regulation of invasion by STAT3 in triple negative breast cancer

SUPPLEMENTARY FIGURES AND TABLES

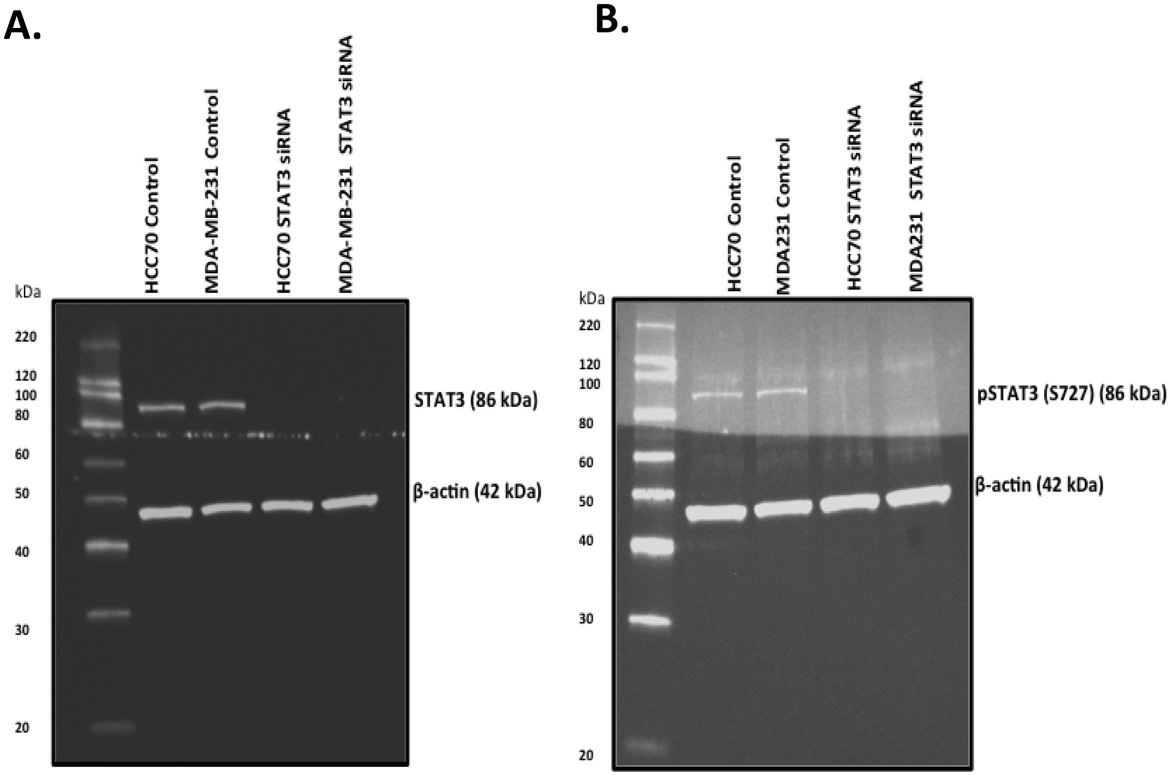

Supplementary Figure S1: Western blot analysis reveals efficient STAT3 siRNA transfection. A. HCC70 and MDA-MB-231 were transfected with STAT3 siRNA or non-targeting siRNA. Knockdown of STAT3 resulted in 97% and 95% STAT3 protein reduction in HCC70 and MDA-MB-231 respectively. **B.** Knockdown of STAT3 resulted in 70% and 60% phosphorylated STAT3 (pSTAT3) protein levels in HCC70 and MDA-MB-231 respectively.

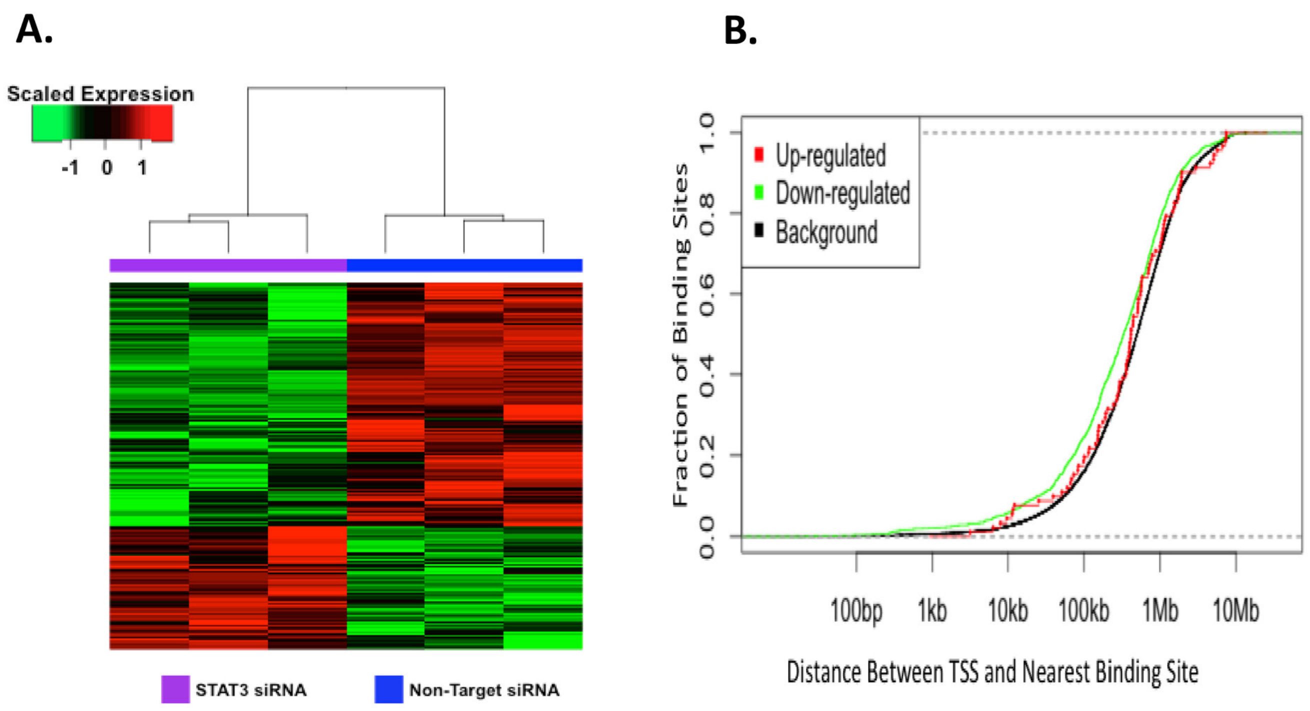

Supplementary Figure S2: Differential expression associated with STAT3 knockdown in HCC1143. A. Heatmap of differential gene expression associated with STAT3 knockdown in HCC1143. 1,732 genes were differentially expressed in response to STAT3 knockdown (> 2.0-fold differences between STAT3 siRNA treated cells and non-targeting vehicle controls). **B.** Cumulative distribution function of STAT3 binding near gene TSS in HCC1143. Genes upregulated and down-regulated upon STAT3 knockdown are significantly enriched with nearby STAT3 binding sites compared to the background genes.

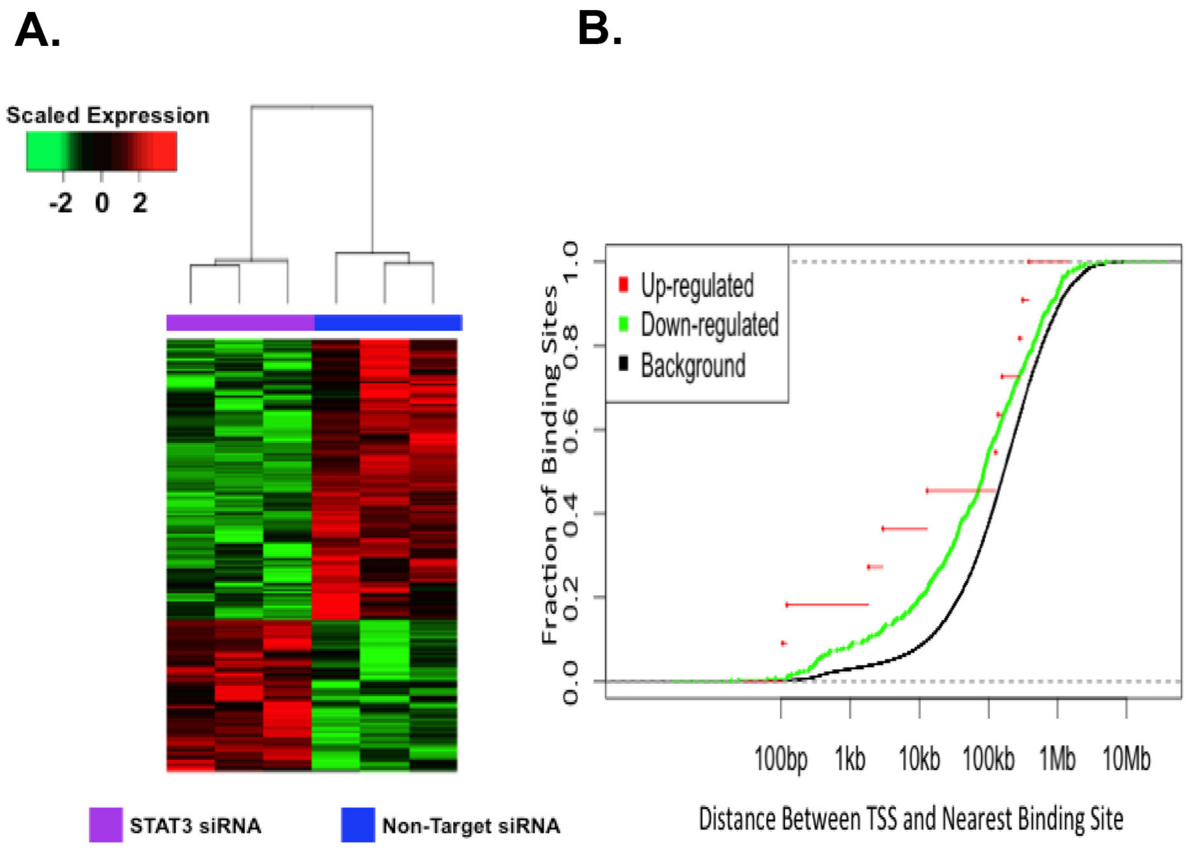

Supplementary Figure S3: Differential expression associated with STAT3 knockdown in MDA-MB-157. A. Heatmap of Differential Gene Expression Associated with STAT3 Knockdown in MDA-MB-157. 546 genes were differentially expressed in response to STAT3 knockdown (> 2.0-fold differences between STAT3 siRNA treated cells and non-targeting vehicle controls). **B.** Cumulative distribution function of STAT3 Binding Near Gene TSS in MDA-MB-157. Genes upregulated and down-regulated upon STAT3 knockdown are significantly enriched with nearby STAT3 binding sites compared to the background genes.

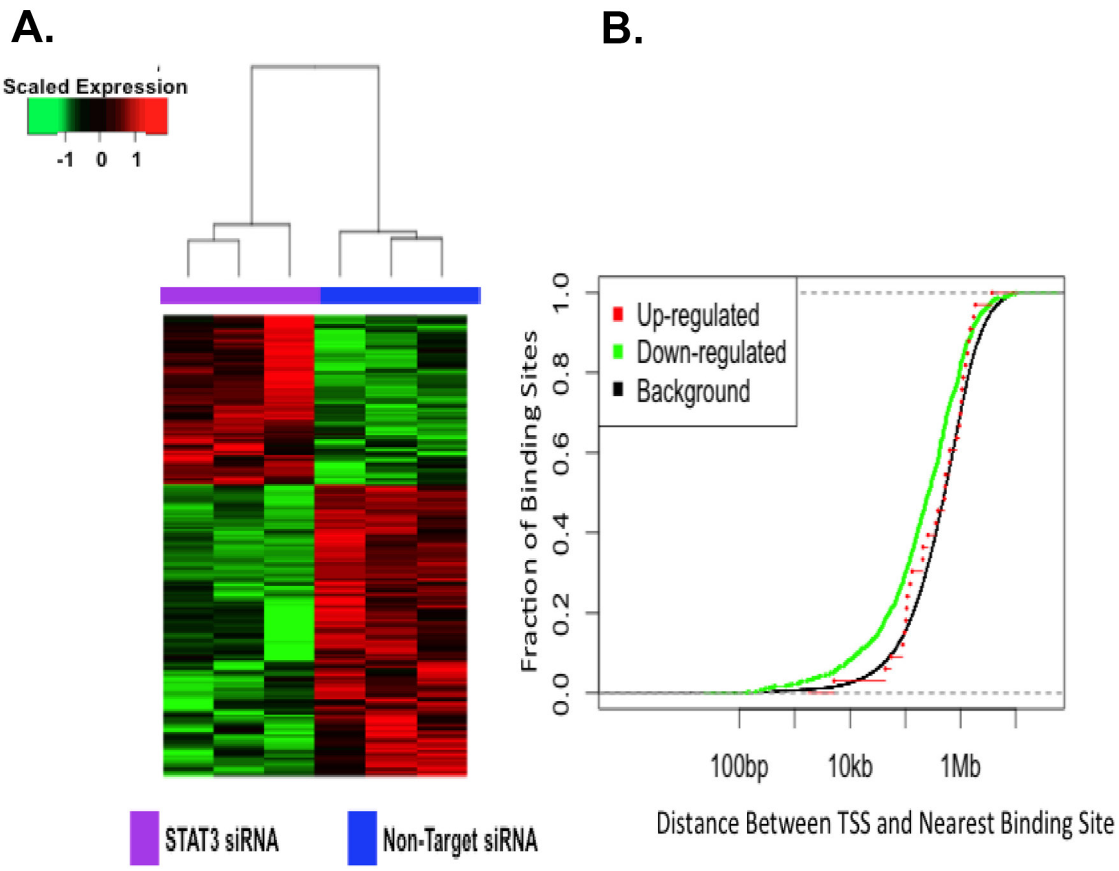

Supplementary Figure S4: Differential expression associated with STAT3 knockdown in MDA-MB-468. A. Heatmap of differential gene expression associated with STAT3 knockdown in MDA-MB-468. 854 genes were differentially expressed in response to STAT3 knockdown (> 2.0-fold differences between STAT3 siRNA treated cells and non-targeting vehicle controls). **B.** Cumulative distribution function of STAT3 binding near gene TSS in MDA-MB-468. Genes upregulated and down-regulated upon STAT3 knockdown are significantly enriched with nearby STAT3 binding sites compared to the background genes.

Supplementary Table S1: MSigDB Perturbations Enriched Near TNBC STAT3 Binding Sites

Enrichment	FDR q-value
Class I of genes transiently induced by EGF in 184A cells (mammary epithelium)	0
Genes up-regulated in T34 (bladder cancer) cells in response to the photodynamic therapy (PDT) stress	0
Down-regulated genes that vary between HNSCC (head and neck squamous cell carcinoma) group formed on the basis of their level of pathological differentiation: well vs poorly differentiated tumors.	0
Genes up-regulated in HL-60 (acute promyelocytic leukemia, APL) after with the aminopeptidase inhibitor tosedostat for 24hr.	0
Genes up-regulated in PC3 cells (prostate cancer) after knockdown of EZH2 by RNAi.	0
Up-regulated genes in colon carcinoma tumors compared to the matched normal mucosa samples.	0
Genes up-regulated in ME-A cells (breast cancer) undergoing apoptosis in response to doxorubicin.	0
Genes whose promoters are bound by MYC according to MYC Target Genes Database.	0
Genes up-regulated in uveal melanoma: class 2 vs class 1 tumors.	0
Genes down-regulated in NB4 cells (acute promyelocytic leukemia, APL) in response to tretinoin based on ChIP-seq data.	0
Genes down-regulated in PC3 cells (prostate cancer) after knockdown of NIPP1 by RNAi.	0
Transcripts depleted from pseudopodia of NIH/3T3 cells (fibroblast) in response to haptotactic migratory stimulus by fibronectin, FN1.	0
Class III of genes transiently induced by EGF in 184A1 cells (mammary epithelium).	0
Genes with promoters bound by FOXP3 in hybridoma cells stimulated by PMA and ionomycin.	0
Genes induced under hypoxia independently of KDM3A in RCC4 cells (renal carcinoma) expressing VHL.	0
Genes up-regulated in confluent IMR90 cells (fibroblast) after knockdown of RB1 by RNAi.	0
Genes with promoters bound by both PPARG and RXRA at 8 day time point of and RXRA at 8 day time point of adipocyte differentiation of 3T3-L1 cells (preadipocyte).	0
Genes up-regulated in MC7 cells (breast cancer) after stimulation with NRG1.	0
Genes commonly down-regulated in human alveolar rhabdomyosarcoma (ARMS) and its mouse model overexpressing PAX3-FOXO1 fusion.	0
Genes down-regulated in luminal-like breast cancer cell lines compared to the basal-like ones.	0

STAT3 binding sites in TNBC cell lines and tumors are near genes expressed in basal-like breast cancer (enrichments determined using GREAT)

Supplementary Table S2: Biological Processes Enriched Near TNBC STAT3 Binding Sites

Enrichment	FDR q-value
Response to Wounding	0
Cardiovascular System Development	0
Regulation of Intracellular Protein Kinase Cascade	0
Vasculature Development	1.73×10^{-280}
Wound Healing	4.29×10^{-274}
Response to Biotic Stimulus	8.57×10^{-271}
Response to Other Organism	1.92×10^{-267}
Blood Vessel Development	5.53×10^{-267}
Response to Cytokine Stimulus	3.64×10^{-260}
Cell Activation	2.64×10^{-241}
Blood Vessel Morphogenesis	1.10×10^{-238}
Regulation of Cellular Component Movement	1.32×10^{-234}
Negative Regulation of Protein Metabolic Process	6.22×10^{-232}
Positive Regulation of Intracellular Protein Kinase Cascade	8.35×10^{-223}
Regulation of Defense Response	1.57×10^{-222}
Regulation of Cell Motility	9.00×10^{-220}
Regulation of Cell Migration	2.97×10^{-218}
Regulation of Locomotion	1.20×10^{-216}
Extracellular Matrix Organization	3.18×10^{-208}
Extracellular Structure Organization	4.36×10^{-208}

STAT3 binding sites in TNBC cell lines and tumors are near biological processes likely to promote cell migration, invasion and metastasis (enrichments determined using GREAT)

Supplementary Table S3: Cellular Processes Enriched Near TNBC STAT3 Binding Sites

Enrichment	FDR q-value
Anchoring Junction	$3.20x10^{-175}$
Actin Cytoskeleton	$9.00x10^{-172}$
Adherens Junction	$8.73x10^{-163}$
Extracellular Membrane-bounded organelle	$3.40x10^{-139}$
Extracellular Vesicular Exosome	$5.24x10^{-134}$
Cell-substrate Junction	$5.33x10^{-133}$
Cell-substrate Adherens Junction	$1.40x10^{-116}$
Focal Adhesion	$2.67x10^{-116}$
Cell Cortex	$1.21x10^{-97}$
Actomyosin	$1.32x10^{-75}$
Intermediate Filament Cytoskeleton	$2.68x10^{-71}$
Stress Fiber	$5.99x10^{-68}$
Actin Filament Bundle	$7.76x10^{-68}$
Ruffle	$7.93x10^{-67}$
Cytosolic Part	$3.00x10^{-64}$
Cell Cortex Part	$2.47x10^{-62}$
Basement Membrane	$3.40x10^{-62}$
Lamellipodium	$1.60x10^{-61}$
Melanosome	$3.78x10^{-61}$
Apical Junction Complex	$2.20x10^{57}$

STAT3 binding sites in TNBC cell lines and tumors are near cellular processes likely to promote cell migration, invasion and metastasis (enrichments determined using GREAT)

Supplementary Table S4: Genes Commonly Regulated by STAT3 in HCC70 and MDA-MB-231 Cell Lines

Gene	Direction of Regulation Upon STAT3 Knockdown
AIM1L	Up-regulated
ANKRD2	Up-regulated
BEAN1	Up-regulated
BTBD19	Up-regulated
C4A	Down-regulated
C4B-AS1	Down-regulated
CD52	Up-regulated
CPA4	Up-regulated
CPT1C	Down-regulated
GDF15	Up-regulated
HLA-DMB	Down-regulated
HMGN1P36	Up-regulated
LCN2	Up-regulated
MGLL	Up-regulated
NANOS1	Up-regulated
NFE2	Down-regulated
NNMT	Down-regulated
SERPINA3	Downregulated
SFTAIP	Up-regulated
STAT3	Down-regulated
TXTEX1D4	Up-regulated
TNFSF15	Up-regulated

Supplementary Table S5: Top 10 GSEA enrichments of differentially expressed genes in response to 96 hour STAT3 knockdown in HCC1143

Signatures Directly Activated by STAT3 in HCC1143 (Down-regulated Upon STAT3 Knockdown)	FDR q-value
HALLMARK_KRAS_SIGNALING_UP	2.57E-12
HALLMARK_TNFA_SIGNALING_VIA_NFKB	1.26E-08
HALLMARK_P53_PATHWAY	1.38E-07
NABA_MATRISOME	1.38E-07
HALLMARK_HYPOXIA	1.47E-06
HALLMARK_EPITHELIAL_MESENCHYMAL_TRANSITION	1.21E-05
HALLMARK_INFLAMMATORY_RESPONSE	1.21E-05
HALLMARK_INTERFERON_GAMMA_RESPONSE	1.21E-05
REACTOME_TIGHT_JUNCTION_INTERACTIONS	1.21E-05
HALLMARK_APOPTOSIS	2.52E-05

Enrichments represent hallmark and canonical pathways in GSEA database

Supplementary Table S6: Top 10 GSEA enrichments of differentially expressed genes in response to 96 hour STAT3 knockdown in MDA-MB-157

Signatures Directly Activated by STAT3 in MDA-MB-157 (Down-regulated Upon STAT3 Knockdown)	FDR q-value
NABA_MATRISOME	5.95E-10
BIOCARTA_COMP_PATHWAY	2.68E-06
NABA_CORE_MATRISOME	1.29E-05
BIOCARTA_CLASSIC_PATHWAY	2.54E-05
REACTOME_REGULATION_OF_COMPLEMENT_CASCADE	2.54E-05
KEGG_COMPLEMENT_AND_COAGULATION_CASCADES	2.54E-05
NABA_ECM_GLYCOPROTEINS	5.86E-05
NABA_MATRISOME_ASSOCIATED	2.24E-04
PID_INTEGRIN1_PATHWAY	4.04E-04
REACTOME_COMPLEMENT_CASCADE	4.56E-04

Enrichments represent hallmark and canonical pathways in GSEA database

Supplementary Table S7: Top 10 GSEA enrichments of differentially expressed genes in response to 96 hour STAT3 knockdown in MDA-MB-468

Signatures Directly Activated by STAT3 in MDA-MB-468 (Down-regulated Upon STAT3 Knockdown)	FDR q-value
NABA_MATRISOME	5.95E-10
BIOCARTA_COMP_PATHWAY	2.68E-06
NABA_CORE_MATRISOME	1.29E-05
BIOCARTA_CLASSIC_PATHWAY	2.54E-05
REACTOME_REGULATION_OF_COMPLEMENT_CASCADE	2.54E-05
KEGG_COMPLEMENT_AND_COAGULATION_CASCADES	2.54E-05
NABA_ECM_GLYCOPROTEINS	5.86E-05
NABA_MATRISOME_ASSOCIATED	2.24E-04
PID_INTEGRIN1_PATHWAY	4.04E-04
REACTOME_COMPLEMENT_CASCADE	4.56E-04

Enrichments represent hallmark and canonical pathways in GSEA database
